# Supplementary figures and images for: Post-transcriptional control drives Aurora kinase A expression in human cancers
Source: PLoS One. 2024 Nov 11;19(11):e0310625. doi: 10.1371/journal.pone.0310625 (PMC11554201; doi:10.1371/journal.pone.0310625)

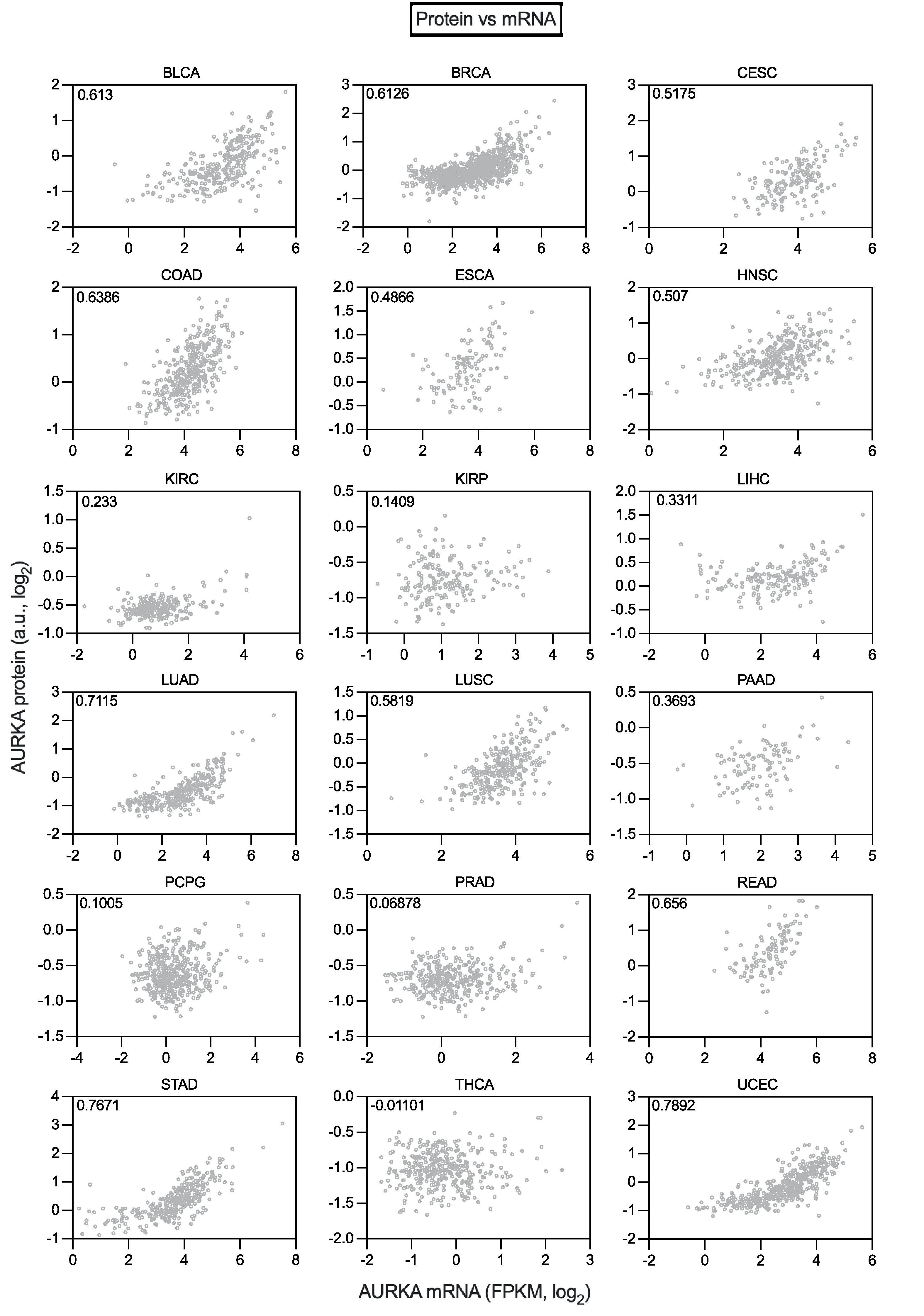

Supplement: S1 Fig — Data points represent individual tumour samples. Values of the Spearman’s rank coefficient (r) shown for each cancer. p<0.001 for all r coefficients except KIRP, PCPG, PRAD and THCA (p>0.05). Note that the ranges of the x and y axes are different in each panel. (TIF) [file pone.0310625.s001.tif]

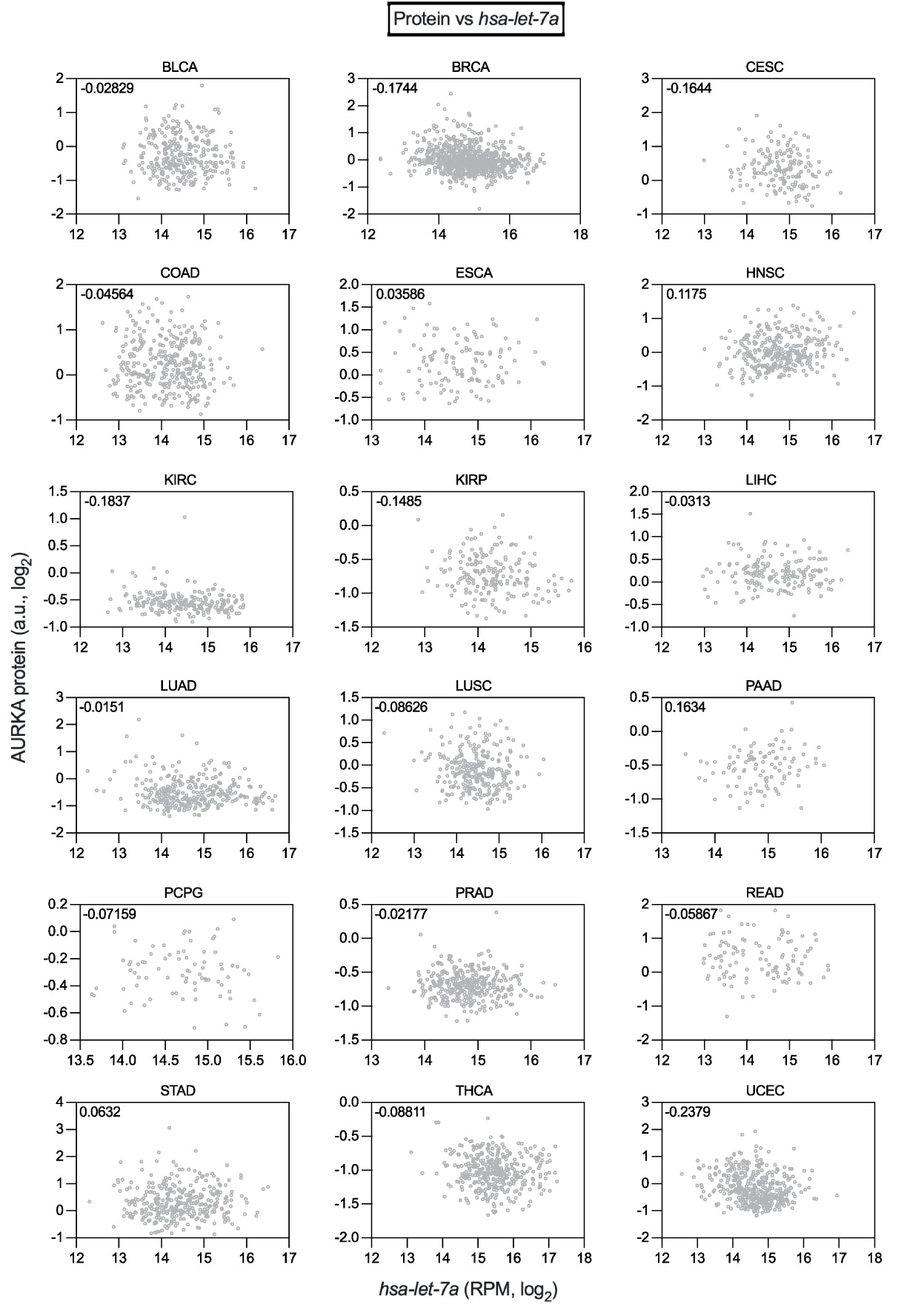

Supplement: S2 Fig — Scatter plots displaying the correlation between expression of AURKA mRNA and hsa-let-7a (A) or AURKA protein and hsa-let-7a (B) in 18 TCGA cancers. Data points represent individual tumour samples. Values of the Spearman’s rank coefficient (r) shown for each cancer. Note that the ranges of the x and y axes are different in each panel. (A) p<0.001 for r coefficients of BRCA, LUAD, PRAD, THCA, UCEC; p<0.05 for r coefficient of LUSC; p>0.05 for all other r coefficients. (B) p<0.001 for r coefficients of BRCA and UCEC; p>0.05 for all other r coefficients. (ZIP) [file pone.0310625.s002.zip › S2_Fig/S2B_Fig.tif]

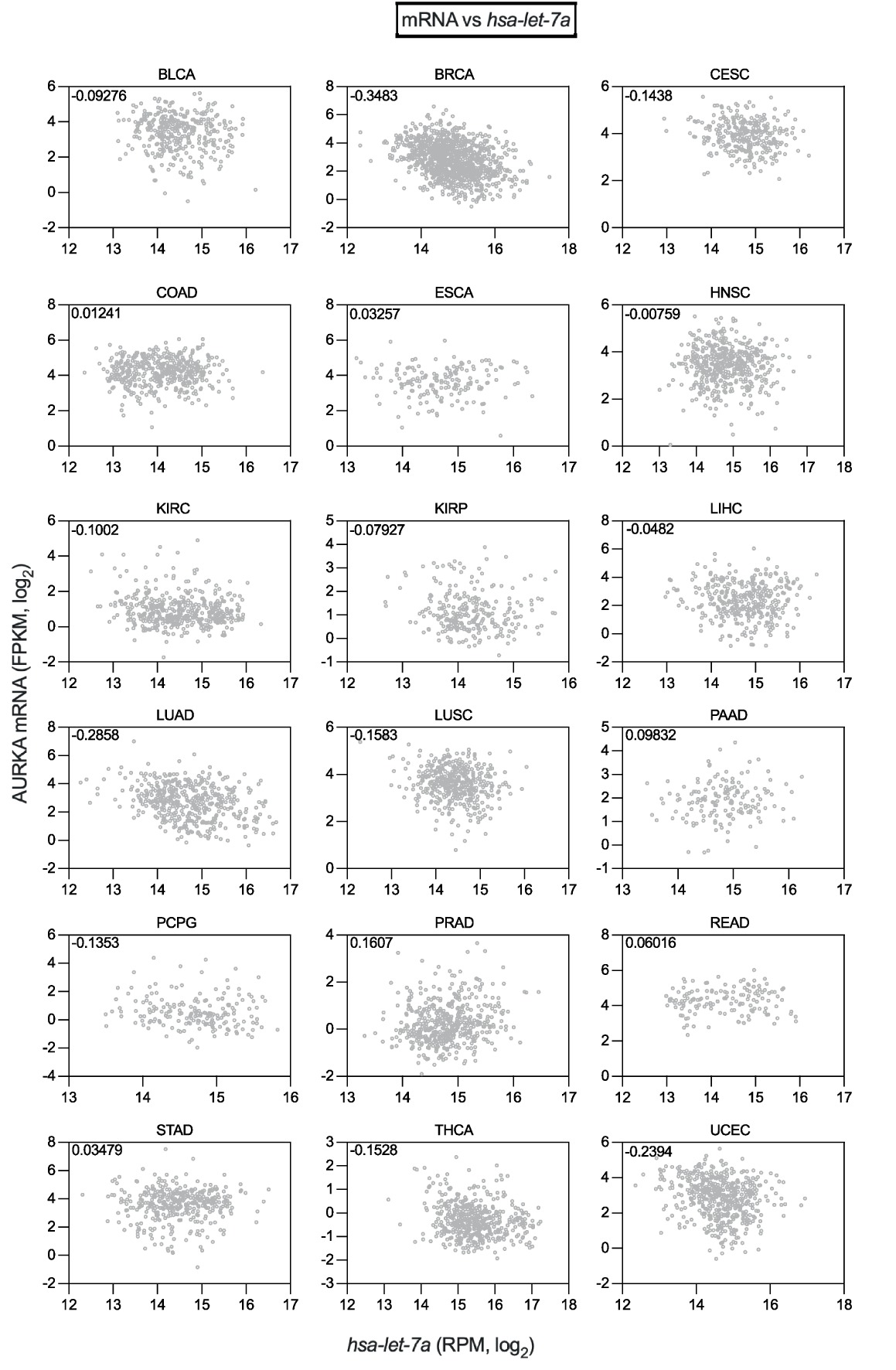

Supplement: S2 Fig — Scatter plots displaying the correlation between expression of AURKA mRNA and hsa-let-7a (A) or AURKA protein and hsa-let-7a (B) in 18 TCGA cancers. Data points represent individual tumour samples. Values of the Spearman’s rank coefficient (r) shown for each cancer. Note that the ranges of the x and y axes are different in each panel. (A) p<0.001 for r coefficients of BRCA, LUAD, PRAD, THCA, UCEC; p<0.05 for r coefficient of LUSC; p>0.05 for all other r coefficients. (B) p<0.001 for r coefficients of BRCA and UCEC; p>0.05 for all other r coefficients. (ZIP) [file pone.0310625.s002.zip › S2_Fig/S2A_Fig.tif]

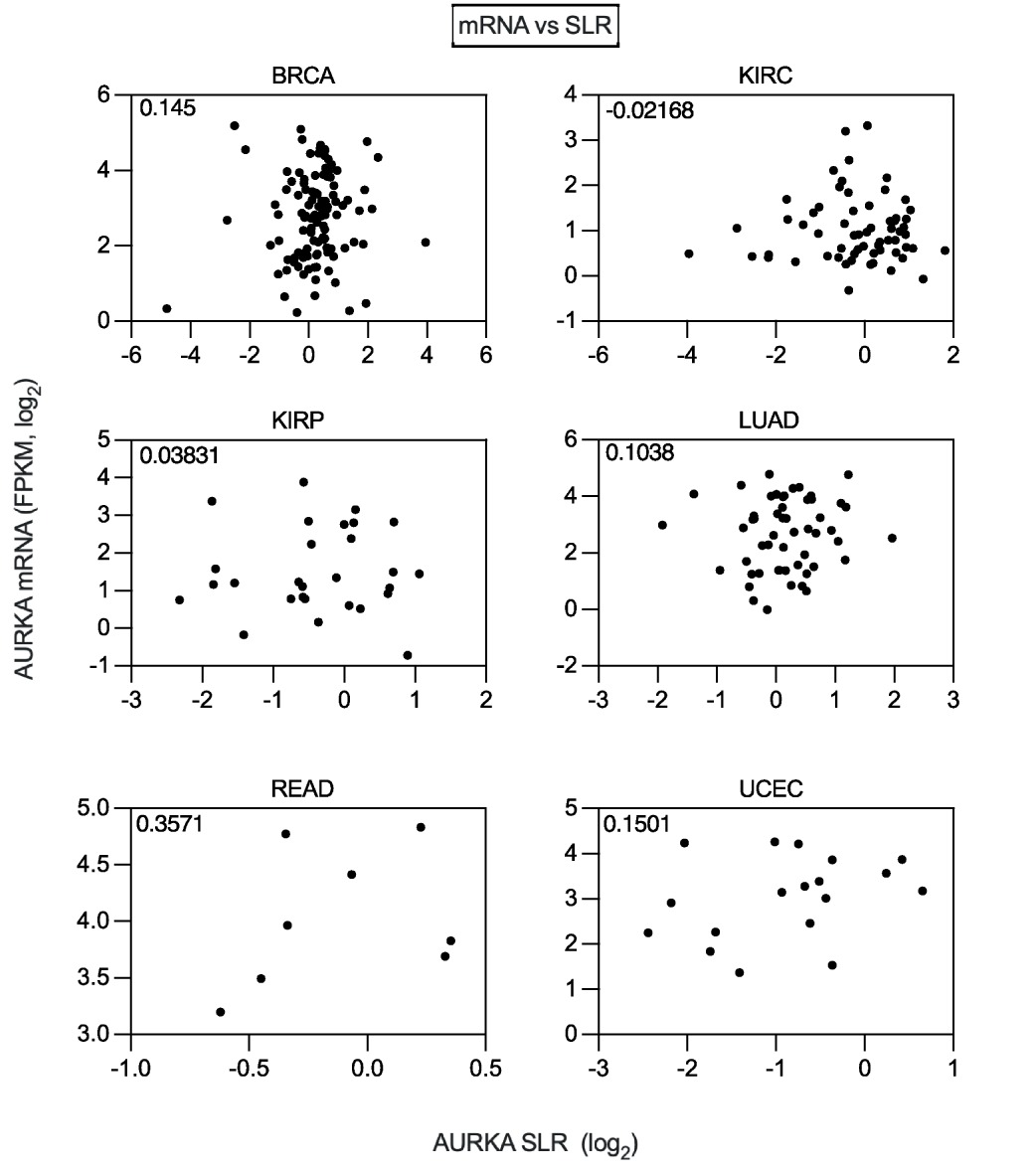

Supplement: S3 Fig — Scatter plots displaying the correlation between AURKA SLR and expression of AURKA protein (A) or AURKA mRNA (B) in selected TCGA cancers. Data points represent individual tumour samples. Values of the Spearman’s rank coefficient (r) shown for each cancer. Note that the ranges of the x and y axes are different in each panel. (A) p<0.05 only for BRCA. (B) p>0.05 for all r coefficients. (ZIP) [file pone.0310625.s003.zip › S3_Fig/S3B_Fig.tif]

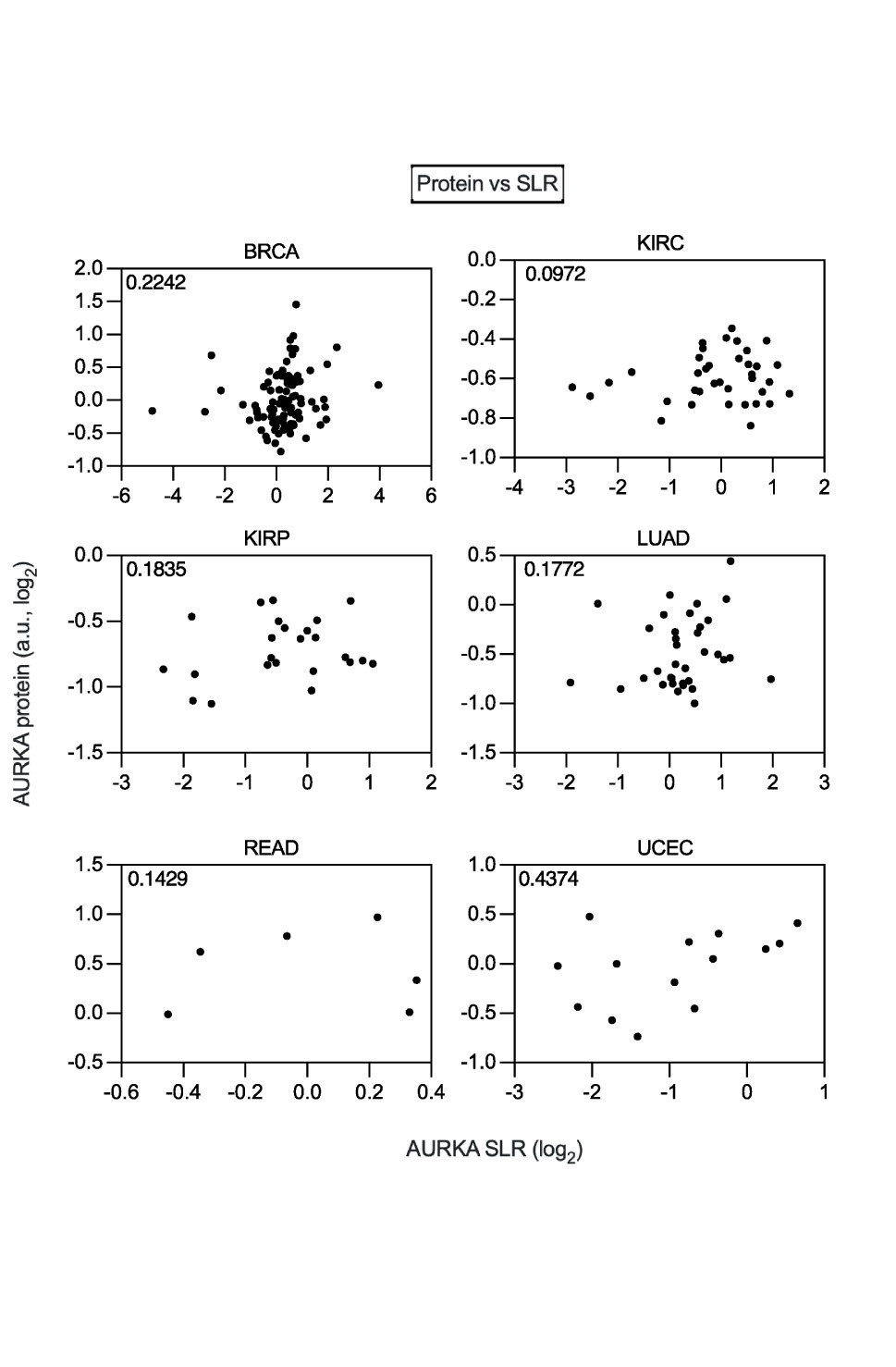

Supplement: S3 Fig — Scatter plots displaying the correlation between AURKA SLR and expression of AURKA protein (A) or AURKA mRNA (B) in selected TCGA cancers. Data points represent individual tumour samples. Values of the Spearman’s rank coefficient (r) shown for each cancer. Note that the ranges of the x and y axes are different in each panel. (A) p<0.05 only for BRCA. (B) p>0.05 for all r coefficients. (ZIP) [file pone.0310625.s003.zip › S3_Fig/S3A_Fig.tif]
