# Supplementary material for: Post-transcriptional control drives Aurora kinase A expression in human cancers
Source: PLoS One. 2024 Nov 11;19(11):e0310625. doi: 10.1371/journal.pone.0310625 (PMC11554201; doi:10.1371/journal.pone.0310625)
Supplement: S1 Script — (PDF) [file pone.0310625.s004.pdf]

```
require(readxl)
require(tidyverse)
library(tidyverse)
library(readr)

#extract datasets as downloaded from TCGA (only files without annotations) and move
into one folder before applying code

setwd()

file_list <- list.files(path="/Protein")
dataset <- data.frame()
for (i in 1:length(file_list)) {
  temp_data <- readr::read_tsv(file_list[i])
  head(temp_data)
  temp_data <- temp_data%>%
    filter(peptide_target=="Aurora-A")%>%
  dataset <- rbind(dataset, temp_data)}
write.csv(dataset,"Protein.csv")

file_list <- list.files(path="/mRNA")
dataset2 <- data.frame()
for (i in 1:length(file_list)) {
  temp_data <- readr::read_tsv(file_list[i], skip = 1)
  temp_data <- temp_data[-c(1,3,4,5,6),]
  head(temp_data)
  temp_data<- temp_data%>%
    filter(gene_name=="AURKA")%>%
  dataset2 <- rbind(dataset2, temp_data)}
write.csv(dataset2,"/mRNA.csv")

file_list <- list.files(path="/let7a")
dataset3<- data.frame()
for (i in 1:length(file_list)) {
  temp_data <- readr::read_tsv(file_list[i])
  head(temp_data)
  temp_data <- temp_data%>%
    filter(miRNA_ID=="hsa-let-7a-1"| miRNA_ID=="hsa-let-7a-2" | miRNA_ID =="hsa-let-7a-
3")%>%
  dataset3 <- rbind(dataset3, temp_data)}
write.csv(dataset3,"/Let7a.csv")


#Match datasets by Sample ID

setwd()

Protein2 <- Protein%>%
  select(c(`Project ID`, `Sample ID`, `Sample Type`, `protein_expression`))
names(Protein2)[names(Protein2) == "protein_expression"] <- "result"
names(Protein2)[names(Protein2) == "peptide_target"] <- "name"
Protein3 <- Protein2
names(Protein3)[names(Protein3) == "result"] <- "aurka_protein_expression"

mRNA2 <- mRNA%>%
  select(c(`Project ID`, `Sample ID`, `Sample Type`, `fpkm_unstranded`))
names(mRNA2)[names(mRNA2) == "fpkm_unstranded"] <- "result"
names(mRNA2)[names(mRNA2) == "gene_name"] <- "name"
mRNA3 <- mRNA2
names(mRNA3)[names(mRNA3) == "result"] <- "aurka_mrna_expression"

Let7a2 <- Let7a%>%
  select(c(`Project ID`, `Sample ID`, `Sample Type`, `reads_per_million_miRNA_mapped`,
`miRNA_ID`))
names(Let7a2)[names(Let7a2) == "reads_per_million_miRNA_mapped"] <- "result"
names(Let7a2)[names(Let7a2) == "miRNA_ID"] <- "name"
Let7a3 <- Let7a2%>%
  group_by(`Sapmle ID`)%>%
  summarise(total_mirnareads=sum(result))
```

```
Let7a4 <- unique(Let7a3)
Let7a5 <- merge(Let7a4, Let7a2, by=c("Sample ID"))


mRNA_Protein <- merge(mRNA3, Protein3, by=c("Sample ID"))
write.csv(mRNA_Protein,"/mRNA_Protein.csv")

mRNA_Let7a <- merge(mRNA3, Let7a5, by=c("Sample ID"))
write.csv(mRNA_Let7a,"/mRNA_Let7a.csv")

mRNA_Protein_Let7a <- merge(mRNA_Protein, Let7a5, by=c("Sample ID"))
write.csv(mRNA_Protein_Let7a,"/mRNA_Protein_Let7a.csv")
```
