# Supplementary material for: Post-transcriptional control drives Aurora kinase A expression in human cancers
Source: PLoS One. 2024 Nov 11;19(11):e0310625. doi: 10.1371/journal.pone.0310625 (PMC11554201; doi:10.1371/journal.pone.0310625)
Supplement: Attachment — Submitted filename: 144319_1_rebuttal_3025480_sc6vvc.docx [file pone.0310625.s008.docx]

**Manuscript number:** RC-2024-02388

**Corresponding author(s):** Roberta, Cacioppo

## 1. General Statements

We would like to thank the reviewers for their attentive reading of our manuscript. We appreciate all the comments and suggestions. We have addressed all the concerns and have included point-by-point responses labeled as blue text in this document.

## 2. Description of the planned revisions

***Reviewer #1***

Minor comments:

1) All of the Correlation analysis have been tested for statistical significance and these results are available in the supplementary data. However, I think it would be useful if these statistics were also included in the main figures themselves (Figures 1B, 2B and 2C). A low correlation that is statistically significant is a more powerful statement.

We agree, and plan to add the results of the statistical analyses in the Figures 1B, 2B and 2C.

2) In the materials and methods, Correlation is separated into distinct degrees: none to very strong, but apart from some lines on the graphs, these degrees of correlation strength are never revisited, so they should be included. Perhaps there is a biological difference between AURKA post transcriptional regulation and protein levels with different R score strength?

We believe that reiterating a discussion on the degrees of correlation strength in the main text would appear repetitive. We do however plan to add a sentence to appropriate points in the main text to redirect the reader to the materials and methods section for information on the distinct degrees of correlation.

3) In Figure 2D a clustering analysis was performed to show the possible relationships between hsa-let-7a and protein levels. The current visualization is hard to understand. A 3D graph with Protein, mRNA and has-let-7a axis's would be easier to follow. I believe it would also be beneficial to do something similar including the APA data as this is the area that the paper lacks depth.

We agree that 3D graphs could aid visualization and plan to provide a link to an interactive 3D view of our analysis.

***Reviewer #2***

Major comments:

2. The statistical significance of the analyses is often unclear. For the correlations between Aurora A protein levels and hsa-let-7a, authors mention that two cancers have a correlation with "statistical significance", but I cannot find any indication of how that was determined, and it is not shown in the corresponding figure (2C). The only time significance is indicated for a correlation is in Figure 4A. Is this the only correlation in the whole manuscript with a p-value less than .05?

The results of the statistical analyses are included in the corresponding supplementary data (Sup. Fig 1, Sup. Fig. 2A-B). We plan to add them to the Figures 1B, 2B and 2C as requested by another reviewer.

3. The SLR for the Aurora A transcripts is only shown in terms of a ratio between cancer and normal tissue. Without the numbers in the absence of normalization, it is difficult to determine how meaningful this is. Is a two-fold change going from .3 to .6 or .001 to .002?

We plan to add a supplementary table containing the SLR values for matched normal and cancer samples in the absence of normalization.

4. Figure 5B is nearly impossible to interpret due to the extreme differences in overall transcript levels between the cancer types. The differences in scaling of the y-axis between the plots makes this even more challenging. The authors state that "It is evident that each isoform has an individual profile of expression across cancers", but this could only be determined from relative expression levels between the different isoforms instead of absolute levels.

We retrieved this plot from the GEPIA2 platform without possibility of editing the y-axis. We plan to edit the text to "It is likely that each isoform has an individual profile of expression across cancers, however a measure of the relative expression levels between the different isoforms would be required”.

Minor comments:

5. In supplementary figure 3, SLR is plotted on a log scale in A and a linear scale in B.

We plan to convert the SLR scale in Sup. Fig. 3B to a log scale.

***Reviewer #3***

Minor comments:

3) Does an alternative AURKA splicing change G1/S to G2/M-phase roles of AURKA? I understand that mRNA is repressed by hsa-let-7a in G1 and S phases but not in G2, so how does non M-phase AURKA protein get made? This may be beyond the scope of the study at this point.

Whether alternative AURKA transcripts change non-mitotic roles of AURKA is an open and intriguing question. In acknowledgement of this point raised by the reviewer, we plan to add a discussion on this in the main text: “Although there is no evidence to date that different AURKA transcripts might influence AURKA activity, instances of isoform-dependent protein localization and function are increasingly reported (Mitschka and Mayr, Nat Rev Mol Cell Biol, 2022). In a previous study, we have detected higher nuclear localization of a reporter protein under the regulation of AURKA short 3’UTR (Cacioppo et al., eLife, 2023). Therefore, there is a possibility that AURKA mRNA isoforms are targeted to different subcellular localizations to support localized translation – or that AURKA protein is co-translationally targeted to different compartments – and AURKA may be preferentially localized in the nucleus when coded by the short 3’UTR mRNA”.

AURKA protein levels are maintained very low in G1 to S phase compared to G2 and M phases. At the level of translation, this is likely ensured by the absence of factors/mechanisms that activate AURKA translation (e.g., hnRNP Q1) and the presence of factors/mechanisms that repress its translation (e.g., hsa-let-7a), the combination of which results in basal translation of AURKA in G1/S until full translational activation in G2 (where a switch likely occurs whereby activating factors operate while repressing factors are disabled). However, the combination and synergy of these factors/mechanisms are likely cell type- and context-dependent.

## 3. Description of the revisions that have already been incorporated in the transferred manuscript

*N/A*

## 4. Description of analyses that authors prefer not to carry out

***Reviewer #1***

Major comments:

1) Unfortunately, there is a major flaw in the TCGA AURKA protein quantification data that underpins much of this study. Following the protein data trail (via https://docs.gdc.cancer.gov/Data/Introduction and its dependents), it appears to rely on the CST anti-AURKA #14475 which is raised to an antigen around Pro70.

We believe the reviewer refers to work from Bertolin et al. 2018 paper (https://doi.org/10.7554/eLife.38111.001) that describes the appearance of truncated versions of AURKA in mitochondrial fractions of cell extracts and shows they depend upon the presence of PMPCB mitochondrial matrix peptidase. We are not familiar with any other literature describing this phenomenon. In our own hands we find AURKA present in the mitochondrial fraction, but the protein is mostly full-length (Grant et al. 2018, https://doi.org/10.1098/rsob.170272). In both papers the mitochondrial pool is small relative to the total cellular pool of AURKA. In fact, this mitochondrial pool is so difficult to detect in intact cells that it has not been reported by other labs and is not universally acknowledged. Given the small size of the mitochondrial pool, any increased amounts of mitochondrial AURKA in cancers, it would be unlikely to significantly impact the measured total protein levels.

2) Following the flaws identified in the protein foundation data, the study would then benefit from some post-validation of findings with actual biological data derived from their own independent assessment of the cancers being examined.

The literature thoroughly reports empirical evidence on AURKA protein expression levels in the cancers analysed in this study, therefore we don’t believe our own post-validation of findings would add any novelty in this sense.

Minor comments:

4) Figure 3B and 3C, can you apply a statistical test on the SLR ratios given the magnitude difference between CCND1 and AURKA SLRs?

Since the values of AURKA and CCND1 SLRs are not always coming from the same dataset and are therefore not matched for patients, we believe it would not be appropriate to make comparisons applying statistical tests.

5) Even though the paper does not claim to provide a unifying hypothesis for APA/has-let-7a regulation of AURKA, I think a more in depth look at the data would be useful. The discussion starts off well when describing what was found with the analysis, but as is, is mostly a re-statement of the results without added insight.

We agree that more in depth analysis of more data would be useful in strengthening conclusions. However, given the variability in interplay between APA and hsa-let-7a we describe, it is well beyond the scope of this study (or the extent of TCGA database) to come up with a unifying hypothesis.

***Reviewer #2***

Major comments:

1. The biggest issue that I have with this analysis relates to the assumption that Aurora A levels will be meaningfully different between individual tumors in all cancer types. For some cancers, the lack of a correlation between mRNA and protein levels for Aurora A could simply be because Aurora A overexpression is not a feature of that cancer type. Looking at the data, the cancer types where they see little-to-no correlation are the cancer types where none of the tumors have high levels of Aurora A mRNA or protein. Therefore, the lack of correlation is likely because differences in protein levels result from noise in the measurements rather than posttranscriptional regulation. Since the lack of correlation between protein and mRNA in these cancer types is the main evidence for the primary conclusion in the paper that "AURKA mRNA and protein expression are often discordant in cancer as a result of dynamic post-transcriptional regulation", I don't think that this conclusion is supported by the data. If anything, the data seems to show that substantial changes in Aurora A protein levels are almost always accompanied by a corresponding change in mRNA levels.

To address this issue, the authors could look at the variability in Aurora A protein levels for each cancer type, and then focus their correlation analyses on cancer types where overexpression of Aurora A is a feature.

We thank the reviewer for this thoughtful comment. We decided not to consider data on AURKA protein levels between healthy and tumour samples because of the lack of proteomic datasets of matching normal tissues for all cancers (except BRCA) in the TCGA database. For this reason, it cannot be excluded that the tumours where we see little-to-no protein-mRNA correlation have in fact high levels of AURKA protein. Indeed, the literature reports wide empirical evidence that AURKA protein is overexpressed in the cancer tissues where we see little-to-no protein-mRNA correlation (Thyroid cancer: Zhao et al, Cell Biosci, 2022; Jingtai et al, Cell Death Dis, 2023. Prostate cancer: Das et al, Pathol, 2010; Chun Yu Lee et al, Cancer Res, 2006. Kidney cancers: Wen et al, Heliyon, 2024; Li et al, Cell Death Dis, 2022. No evidence available for PCPG). Therefore, we believe that is reasonable to propose that in these cancers, which according to our analysis of TCGA data only show minor or no increase in AURKA mRNA expression compared to the normal tissue, lack of correlation is because of post-transcriptional regulation.

Minor comments:

6. Figure 4D is a correlation of correlations. I don't see how to interpret this in a meaningful way.

Figure 4D is not intended for quantitative analysis of correlation of correlations (no quantitative coefficients were in fact calculated), rather to visualize how the link of AURKA SLR with AURKA protein levels and that with hsa-let-7a levels can be differently associated in different cancers.

***Reviewer #3***

Minor comments:

1) To strengthen the study, some analysis of AURKB mRNA would be useful in the same datasets, because this is also an M-phase kinase.

We carried out a specific study of AURKA (and to some extent also of the cell cycle regulator CCND1) using time-limited access to private TCGA datasets. Although we agree that investigation of AURKB would potentially enable us to strengthen some conclusions, this would be a new project that we do not currently have resources for.

2) What happens to TPX2 or CEP192 mRNA (splicing or levels) in the same samples? For TPX2 in particular, this is described in the literature to help form the oncogenic holoenzyme, as well as dictating AURKA protein stability.

Again, we like this suggestion but are not in a position to carry out analyses of TPX2 and CEP192 within the scope of this study.
